# Exploration of barriers and enablers to diabetes care for Aboriginal people on rural Ngarrindjeri Country

**DOI:** 10.1002/hpja.915

**Published:** 2024-08-28

**Authors:** Shanti Omodei‐James, Annabelle Wilson, Renee Kropinyeri, Darryl Cameron, Sharon Wingard, Caitlin Kerrigan, Talia Scriven, Stacy Wilson, Amy E. Mendham, Brooke Spaeth, Stephen Stranks, Billingsley Kaambwa, Shahid Ullah, Paul Worley, Courtney Ryder

**Affiliations:** ^1^ College of Medicine and Public Health Flinders University Adelaide Australia; ^2^ Flinders Health and Medical Research Institute Flinders University Adelaide Australia; ^3^ Riverland Mallee Coorong Local Health Network South Australia Health Adelaide Australia; ^4^ Moorundi Aboriginal Community Controlled Health Service Murray Bridge Australia; ^5^ Coorong Medical Centre Meningie Australia; ^6^ Southern Adelaide Diabetes and Endocrine Services South Australia Health Adelaide Australia; ^7^ The George Institute for Global Health University of New South Wales Sydney Australia; ^8^ School of Population Health University of New South Wales Sydney Australia

**Keywords:** co‐design, diabetes mellitus type 2, Indigenous peoples, knowledge Interface methodology, qualitative research, rural health

## Abstract

**Issues Addressed:**

Addressing the disproportionate burden of type 2 diabetes prevalence in Aboriginal communities is critical. Current literature on diabetes care for Aboriginal people is primarily focused on remote demographics and overwhelmingly dominated by Western biomedical models and deficit paradigms. This qualitative research project adopted a strengths‐based approach to explore the barriers and enablers to diabetes care for Aboriginal people on Ngarrindjeri Country in rural South Australia.

**Methods:**

Knowledge Interface methodology guided the research as Aboriginal and Western research methods were drawn upon. Data collection occurred using three yarning sessions held on Ngarrindjeri Country. Yarns were transcribed and deidentified before a qualitative thematic analysis was conducted, guided by *Dadirri* and a constructivist approach to grounded theory.

**Results:**

A total of 15 participants attended the yarns. Major barriers identified by participants were underscored by the ongoing impacts of *colonisation.* This was combated by a current of *survival* as participants identified enablers to diabetes care, namely a *history of healthy community*, *working at the knowledge interface*, *motivators* for action, and an abundance of *community skills and leadership*.

**Conclusions:**

Despite the raft of barriers detailed by participants throughout the diabetes care journey, Aboriginal people on Ngarrindjeri Country were found to be uniquely positioned to address diabetes prevalence and management.

**So What?:**

Health promotion efforts with Aboriginal people on Ngarrindjeri Country must acknowledge the sustained impacts of colonisation, while building on the abundance of community enablers, skills and strengths. Opportunities present to do so by adopting holistic, community‐led initiatives that shift away from the dominant biomedical approach to diabetes care.

## 1 INTRODUCTION

Type 2 diabetes is predicted to become the largest epidemic in human history.^1^ For Aboriginal and Torres Strait Islander people,^1^ the financial and human cost of this epidemic is well known as communities continue to disproportionately bear the burden of this chronic disease.^2^ Prior to colonisation there was little evidence of diabetes prevalence in Australia.^3^ Colonial policies generated inherently complex and arguably traumatic relationships with food and nutrition for Aboriginal people, as controlled access to food and medicine was used to enforce assimilationist values and colonial social norms.^4^ Ongoing colonisation has been additionally shown to act adversely on the protective mechanisms embodied in the cultural determinants of Aboriginal health, resulting in inequitable health outcomes.^5^

The diabetes care experience has been linked to social and cultural determinants of health.^6^, ^7^ For instance, socioeconomic position has been found to influence the risk of mortality and diabetes‐related complications.^8^ Considering the position maintained by Aboriginal communities across numerous socioeconomic indicators,^9^ it is unsurprising that Aboriginal and Torres Strait Islander people continue to experience diabetes diagnosis and mortality rates at three and five times the rate of non‐Indigenous people respectively.^10^ The prevention and management of diabetes in Aboriginal communities is critical.^2^

In response to these sustained inequitable health outcomes, the most recent Australian National Diabetes Strategy and peak Aboriginal research bodies increasingly recognise the need to adopt strengths‐based approaches, tailor interventions to suit local community needs and partner with communities to promote self‐determination.^10^, ^11^, ^12^, ^13^ To date however, approaches to diabetes care with Aboriginal people continue to be dominated by biomedical and behaviourist models, promoting a discourse of deficit and portraying Aboriginal people through a lens of difference, disparity, disadvantage, dysfunction and deprivation.^14^, ^15^ Aboriginal people are primarily represented as lacking the necessary skills and knowledge to manage diabetes care, as reflected in the dominance of interventions focusing on nutrition education, health promotion and lifestyle change.^16^, ^17^ Within this discourse, Western positivist epistemology and ontology remains privileged and invisible as the epistemological a priori.^18^, ^19^ The resulting effect is the continued portrayal of Aboriginal ways of knowing, being and doing as inferior and irrational.^20^

Encouragingly, a growing body of literature has begun privileging the lived experiences of Aboriginal people throughout the diabetes care journey, however this research primarily focuses on remote demographics.^16^ A paucity of qualitative studies have explored Aboriginal diabetes care journeys in rural Australia.^6^, ^21^ While diabetes interventions have been implemented for Aboriginal people in rural South Australia,^22^, ^23^ no qualitative studies prioritising the narratives of these people have occurred. This research sought to fill this gap.

### 1.1 The CDC project

This research emerged out of a larger Medical Research Future Fund (MRFF) grant to co‐design a diabetes and metabolic syndrome intervention with Aboriginal peoples living on Ngarrindjeri Country.^17^ The larger research project was initiated by the Coorong Diabetes Collaborative (CDC) who, in conjunction with Ngarrindjeri Elders, identified the need for a locally driven, community‐designed diabetes intervention. The CDC comprises of representatives from Flinders University, Moorundi Aboriginal Community Controlled Health Service (ACCHS), Coorong Medical Centre and Riverland Mallee Coorong Local Health Network (RMCLHN). The CDC project is governed by a Steering Committee that consists of Aboriginal community representatives living on Ngarrindjeri country.

The first phase of the CDC project, and the corresponding primary aim of this research was an exploration of the diabetes care journey for Aboriginal people living on Ngarrindjeri Country. Adopting a strengths‐based approach, we sought to prioritise Aboriginal narratives and highlight protective factors throughout. Two guiding research questions were developed to achieve these aims.

1. What are the enablers to diabetes care for Aboriginal people on Ngarrindjeri Country?
2. What are the barriers to diabetes care for Aboriginal people on Ngarrindjeri Country?

## 2 METHODS

**FIGURE 1:**
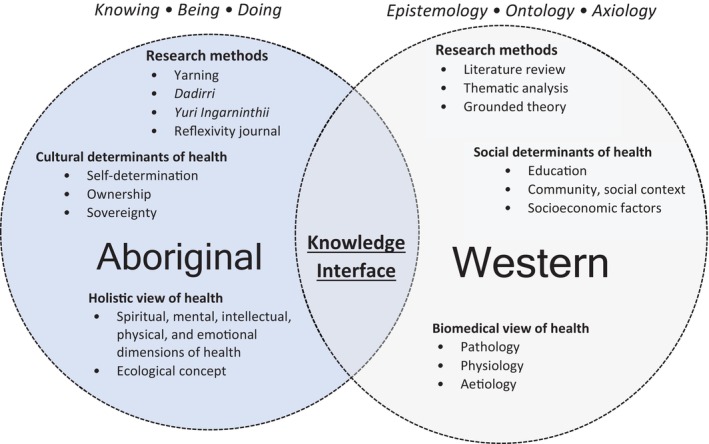
Theoretical framework of the research (adapted from Ryder et al.^28^). Aboriginal knowledge systems are guided by Aboriginal ways of knowing, being and doing and include the methods of yarning, *Dadirri, Yuri Ingarninthii* and reflexivity. Western knowledge systems are guided by traditionally positivist epistemology, ontology and axiology and include the methods of literature review and thematic analysis. The central third space is where these knowledge systems are woven together, the knowledge interface, and is where research findings emerge.

This exploratory study, along with the broader CDC project, adopted Knowledge Interface methodology. In doing so, the strengths of both Aboriginal ways of knowing, being and doing, and Western research epistemology, ontology and axiology were drawn upon.^24^, ^25^ Indigenous and Western knowledge systems worked together, in equal partnership, woven together to produce novel and positive outcomes for both systems.^24^, ^26^ A detailed account of how the student researcher responsible for analysis (SOJ) upheld the core values of working at the interface is provided in Supplementary Table 1.^27^ As demonstrated in Figure 1, methods were directly informed by Knowledge Interface methodology.

### 2.1 Setting

This study took place on Ngarrindjeri Country, across the townships of Meningie, Murray Bridge and Raukkan. Now home to an Aboriginal township, Raukkan was formerly the Point McLeay Mission, abolished in 1974. Ngarrindjeri nation encompasses the area along the Lower Murray, Coorong and Lakes area of South Australia and consists of 18 *Laklinyeris* (tribes). Ngarrindjeri people possess a strong connection to their *Yarluwar‐Ruwe* (Sea Country) and recognise the interconnectedness of all living things.^29^ Ngarrindjeri nation boasts a strong history of community‐led initiatives and political activism to protect *Yarluwar‐Ruwe* and community.

### 2.2 Recruitment

Participants were selected using purposeful sampling, with recruitment efforts focusing on Aboriginal adults living on Ngarrindjeri Country with lived experience of diabetes. Recruitment was facilitated by members of the RMCLHN Aboriginal Health Service and Moorundi ACCHS. Diabetes yarning sessions were advertised through community newsletters, email and posters, allowing prospective participants to join on a voluntary basis.

### 2.3 Data collection

Throughout May of 2023, the three townships were each host to one yarning session. Participants were provided transport by RMCLHN staff as required. Yarns took place at local community centres. Participants were informed of the research aims, questions and the broader purpose of the CDC study.

Ethics for this research was granted through the Aboriginal Health Research Ethics Committee, the RMCLHN and Flinders University. In consideration of data sovereignty principles, the CDC Steering Committee oversaw data collection, analysis, interpretation and dissemination of results. Participants had the opportunity to discuss the research, including the storage and use of participant qualitative data, prior to providing informed, written consent. Participants were compensated with a $30 Visa voucher.

Yarning sessions were run by a Ngarrindjeri researcher (DC) and a non‐Indigenous clinician, known and trusted by community (PW). Derived from Aboriginal ways of knowing, being and doing, yarning is an informal conversational process that involves the mutual sharing of stories and the development of relationships of reciprocity and accountability between yarn participants.^30^ As agreed upon by the CDC Steering Committee, only researchers and RMCLHN staff with a prior relationship to community would attend the yarns. Accordingly, the student researcher (SOJ) was not present. Set questions were not prepared as to allow participants to share their story in their own words and pace, unconfined to the research agenda.^30^ Yarns lasted approximately 1 hour. Local protocols were followed as each yarn began with a relational introduction of self and concluded with the sharing of a healthy meal.

### 2.4 Analysis

Access to audio recordings and deidentified transcripts enabled SOJ to complete the thematic analysis. A constructivist approach to grounded theory guided the analysis, as SOJ conducted a reflexive examination of how knowledge hierarchies informed her as a white, middle‐class, student public health researcher.^31^, ^32^ Efforts by SOJ to adhere to the principles of constructivist grounded theory, contest power imbalances and avoid the historically extractive nature of research, are contained within a reflexivity journey provided in Supplementary Table 2. This constructivist approach complements the values championed by decolonising research, strengths‐based approaches and ensured Aboriginal voices and epistemologies remained central throughout the research process.^18^, ^31^, ^32^, ^33^

Qualitative analysis software Nvivo 1.7 facilitated thematic coding, guided by Ezzy's three steps of open, axial and selective coding.^34^ Prior to commencing the analysis, SOJ utilised the Aboriginal research method of deep listening, to assess the mood, emotions and nuances of the yarn. This method is known to the Ngangikurungkurr people of the Daly River as *Dadirri*, while to the Kaurna people of the Adelaide Plains, it is known as *Yuri Ingarninthii* and requires one to listen intently in an enquiry by the ears.^35^ *Dadirri* was practised by SOJ as she listened to the recordings, free from judgement or pre‐disposition in an exercise of quiet reflection from the heart.^36^ Although time consuming, *Dadirri* proved pivotal to obtaining rich understandings of the data that were layered, detailed and nuanced, as deeper meanings were obtained from recordings and transcripts with each investigation.^37^

Initial interpretations were checked for accuracy with the attending university researchers (CR, AW) and contextual points (pauses, participant tone and body language) were shared to strengthen the analysis. Regular reflective yarning occurred amongst the team and deep listening was employed as researchers moved ‘beyond the surface, searching and questioning for tacit meanings about values, beliefs and ideologies’, before codes were organised around the central theme.^32^ ^(p. 12)^

A series of thematic maps were produced and refined, guided by the narratives of participants. Member checking occurred with attending Aboriginal researchers (DC, SW, RK, TS, SW) and yarn participants. Results were repatriated back to community using infographics during subsequent phases of the CDC study.

## 3 RESULTS

Three yarning sessions were attended by a total of 15 unique participants, with only one participant attending across multiple yarns. Of the participants that consented to sharing demographic data, 10 identified as Elders, five identified as men and the remaining as women. All participants had been diagnosed with diabetes, identified as Aboriginal, and currently lived on Ngarrindjeri Country. One participant was a member of the CDC and a representative of the RMCLHN.

**FIGURE 2:**
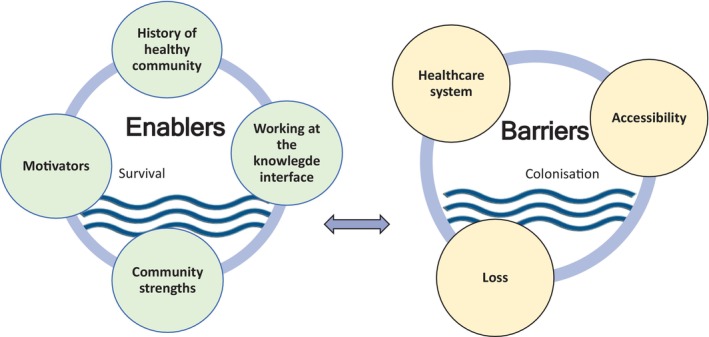
Thematic map of major barriers and enablers to the diabetes care journey for Aboriginal people living on Ngarrindjeri Country. The arrow represents a line of influence, demonstrating that barriers and enablers are interdependent. Circles were used to indicate major themes were similarly connected with one another. The themes of *Survival* and *Colonisation* are represented by waves as the currents underpinning enablers and barriers respectively. Waves were selected as they correspond to the Ngarrindjeri connection to water.

**FIGURE 3:**
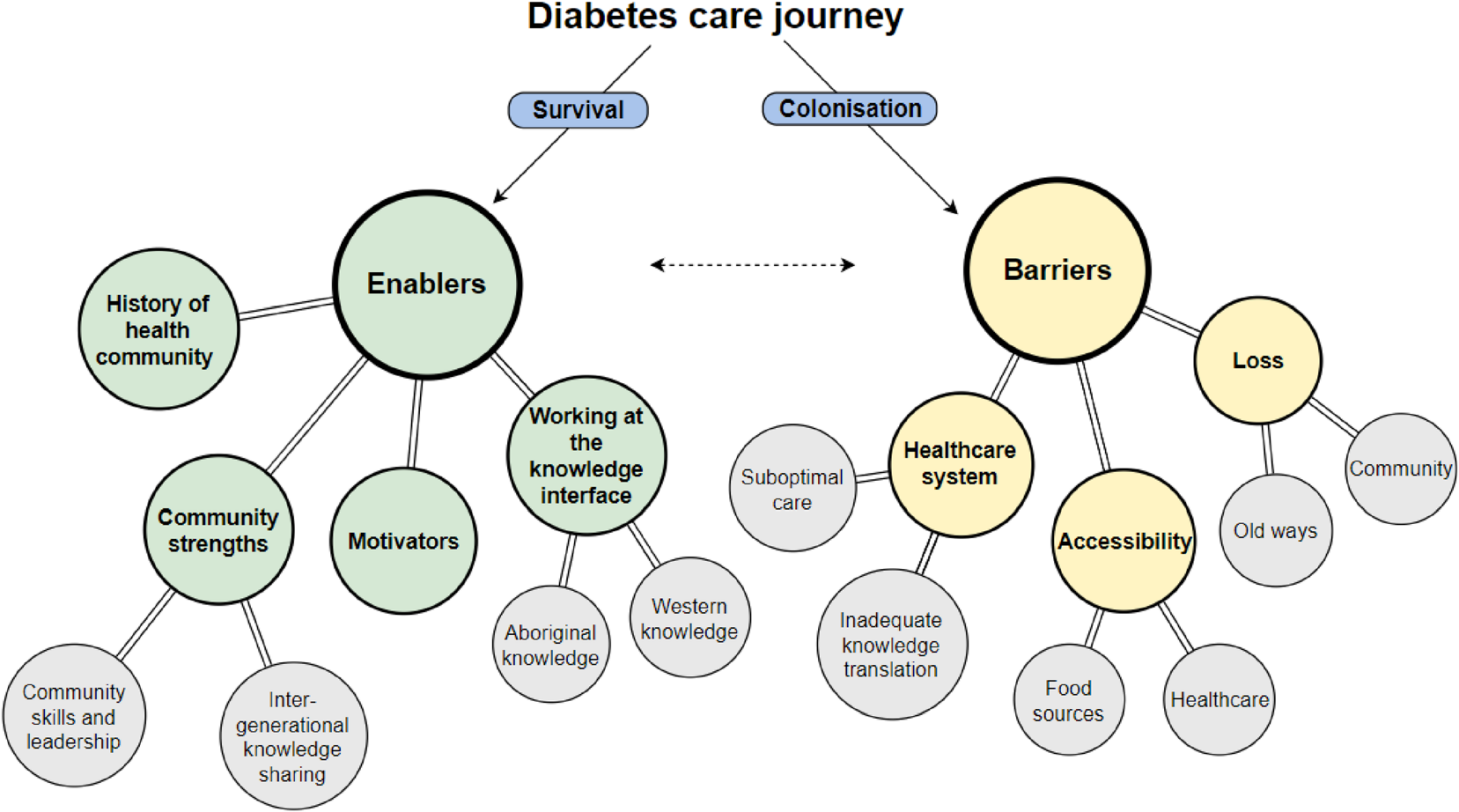
Coding tree of major themes, subthemes and currents. Green circles correspond to the four major enablers while yellow circles correspond to the three major barriers. Grey circles dictate subthemes. *Survival* and *colonisation* underpin enablers and barriers respectively as currents. The arrow represents a line of influence, demonstrating that barriers and enablers are interdependent.

Figure 2 provides an overview of the major barriers and enablers to the diabetes care journey identified by participants. A coding tree was further developed to include details of subthemes and currents, see Figure 3. These images guide the discussion of results.

### 3.1 Enablers

Participants identified four major enablers to their diabetes care journeys. They acted at both the community and individual level.

#### 3.1.1 History of healthy community

By reflecting on childhood experiences growing up on Ngarrindjeri Country, participants identified a history of healthy community and longevity. Guided by ‘the old ways’ (P4) of knowing, being and doing, regular exercise and the consumption of fresh produce from Country was commonplace pre‐colonisation. The health of community was attributed to these old ways:

We weren't sick. We never had a cold. We were running around barefoot out in the rain and everything. (P4)

Sharing food, caring for Country and for each other, were similarly recognised as aiding community health and wellbeing. Meanwhile, the current high prevalence of diabetes was framed as a relatively new phenomena with diabetes an introduced illness:

…growing up down in Raukkan, you know. I didn't know of anybody down Raukkan way who had diabetes. (P10)

#### 3.1.2 Working at the knowledge interface

Participants demonstrated dual understandings of Western biomedical approaches to diabetes care and Aboriginal knowledge of Country, health and food sources. In doing so, participants revealed their ability to work at the interface of Western and Aboriginal knowledge systems.

##### Western knowledge system

Awareness of how lifestyle factors, such as weight, exercise and healthy diet, can impact the diabetes care journey was exhibited. Participants shared their concerns over the availability of fast‐food chains and discussed efforts to avoid salty and sugary foods. Proficiency with diabetes self‐management was demonstrated as participants recognised healthy blood glucose ranges, hypo and hyperglycaemia and discussed the administration of insulin.

So when I'd get home I'd check my sugar and… I write it down every day in my little book I've got… It was 7.6 so I knew I wasn't having a hypo.(P10)

Moreover, symptoms of diabetes were described, including dizzy spells, numbness, changes in vision, blindness or skin tags. Links to comorbidities, namely heart and kidney disease, were also addressed.

##### Aboriginal knowledge system

Discussion of cultural knowledge and practices was common. Participants shared their observations of recent environmental changes to Country, while providing details of how to source and prepare traditional food sources, such as kangaroo, plover, cockles and pig face. Country was described as both teacher and provider. Paying attention to Country and the seasonality of produce was key.

Well, I remember what Mum used to say that, you know, everything was from the land. Yeah, and all of those days it was all about Coorong. So they lived off, you know, the cockles and everything and different things off the Coorong there. (P7)

The health of Ngarrindjeri Country was linked to the health of its people, as encapsulated in the following passage:

Well, even the swans, they don't know when to land. They, ah, all year round, really. Their climate has changed—They're confused. (P2)

Yeah, yeah. They're confused. Our bodies are confused too. (P15)

Participants demonstrated an ability to draw from both Western and Aboriginal knowledge systems as they explored how Aboriginal food sources have been utilised in Western dishes. For example, suggestions were made to use kangaroo meat in spaghetti Bolognese or plover eggs in cakes.

#### 3.1.3 Community strengths

Community strengths were reflected in the practice of intergenerational knowledge sharing and diverse community skills and leadership.

##### Intergenerational knowledge sharing

Traditions of intergenerational knowledge sharing were multi‐directional as Elders indicated they were motivated to share, receive and action knowledge of Country and diabetes care. A strong desire to uphold this tradition and mentor future generations was observed, ‘let us in there and talk to these younger ones, you know’ (P1). Intergenerational knowledge sharing also occurred in discussions of family lineage. Such knowledge was suggested to play a role in community healing as family sought reconnection.

##### Skills and leadership of community

Community skills and leadership were evidenced in references to the Raukkan community council and the Moorundi ACCHS. There was pride in the Ngarrindjeri people and the community's proactive approach to addressing health concerns.

Yeah, but the health service wasn't really here then and that now it's good. I think it's great that we've got what you mob are doing. You know? Because I'm part of it. (P11)

Existing community cooking and gardening skills were also recognised. Participants expressed a desire for community to be cared for *by* community, with skills and labour sourced locally.

#### 3.1.4 Motivation

At the community level, participants were motivated to protect future generations from developing similar diabetes outcomes, citing existing high prevalence rates. Severing the cycle of grief and loss was paramount. Meanwhile, a family history of diabetes and fear of diabetes related comorbidities or complications, including amputations, kidney or heart disease, were strong individual motivators for efficient self‐management and healthy lifestyle adoption.

Well, my dad had two or three heart attacks before he died. So I'm always – and my sister's shunt with her heart. So I would just like to come and get my heart checked every three to four years, whenever, you know? Just to make sure that, um, you know, my heart is okay. (P12)

The role played by family supports to improve exercise and eating habits was also cited as a catalyst for individual behaviour change.

### 3.2 Barriers

Barriers to diabetes care were grouped into three major themes.

#### 3.2.1 Healthcare system

Experiences in the dominant Western biomedical healthcare system impeded participants throughout their diabetes care journeys.

##### Inadequate knowledge translation

Participants reported healthcare professionals did not take the time to sufficiently explain diabetes *with* them, leading to confusion about the diagnosis and frustration regarding their overall healthcare experience.

I've been to that diabetes educator. They tell you the same thing over and over. No. I'm not coming back anymore if you tell me the same thing over and over. (P10)

It was suggested that the ineffective knowledge translation was partially due to the time restraints of General Practitioners (GPs). There was also consensus that diabetes was over‐medicalised, as participants felt GPs would more quickly prescribe mediations than provide them with the resources for non‐medicated self‐management. When medication was prescribed, it was poorly explained to participants:

How come I've got to take this one or that one? I—I'll wait and see [the] Dr but there are eight little ones I take in the morning and three big ones. I'll be rattling later on. Like you know, I'm thinking, I'm going to ask her, “can you tell me which it—what each tablet can do?” (P10)

##### Suboptimal care

Participants additionally raised concerns about a lack of continuity of care and how this impacted medication management and delays in diagnosis. Links to healthcare accessibility were made in conversations about high GP turnover and the irregularity of GP visits to Ngarrindjeri Country. Experiences of being dismissed or racially profiled by clinicians were identified. When diagnosis did occur, participants flagged that referrals or follow‐ups with healthcare providers did not always eventuate.

…one week you'll see a doc and then the next week it's a different doctor and you change your drugs. (P8)

Inadequate knowledge translation, perceived over‐prescription of medication and suboptimal care, left participants with self‐management fatigue and a growing distrust of healthcare professionals.

So she'd had, you know, 20 tablets that she had to take, and she was a bit over it, to be honest. She never used to take her lunchtime ones because she was a bit over taking all these tablets. (P14)

The culmination of these healthcare system barriers places participants at risk of complications from medication fatigue and inefficient diabetes self‐management due to poor knowledge translation.

#### 3.2.2 Accessibility

##### Accessibility of food sources

Accessing the supermarket, identified as the primary access point for food, was challenging with public transport an issue, particularly ‘since our bus disappeared’ (P9). Closure of the local corner shop at Raukkan further compounded food accessibility concerns.

Attending the supermarket was presented as a fortnightly activity linked to payday. Participants cited cost as influencing their choice of food while awareness of which shops housed the cheaper prices was presented as common knowledge during the second yarn. Participants touched on how their socioeconomic position and government policies impacted on the types of food they would eat growing up, often consuming the cheaper cuts of meat such as tripe or offal.

Participants flagged the current accessibility and allure of fast food for the next generation as a concern.

The grannies just run off, they want to go to KFC or McDonald's or whatever. (P10)

Them things [picking fruit or fishing], you don't see them anymore. Like I said, people prefer the fish and chip shop. (P1)

Distinctions were made between food sourced from Country and Western food, thought of as excessively convenient, sweet and unhealthy.

##### Accessibility of healthcare

Access to primary healthcare was a significant barrier during participants' diabetes care journeys. GPs would seldom attend Raukkan and would do so inconsistently. Moreover, participants alluded to the high turnover of healthcare professionals in community:

Well, there used to be one [doctor] living here…So that worked good for all of us here. But that's only when I was a teenager. But after that there was like nothing and just one or two would come and then they're gone and then, you know. (P7)

Participants also expressed frustration that funding for healthcare, local government and infrastructure, was ultimately drying up.

#### 3.2.3 Loss

The theme of *Loss* consistently emerged across all three yarns and comprised of two components: loss of community and loss of the old ways.

##### Loss of community

A sense of profound loss for human life was apparent as yarns regularly included talk of upcoming or recent funerals. Participants expressed their deep and personal sorrow, generating a tone of grief for the yarns. P7 reflected, ‘well, they've been lots of them that are dying young, eh? Even more on the way’.

Loss of community was also observed by way of the erosion of community values. Participants commented on the disappearance of the collective responsibility for one another's family. Changes were reported socially, in technology and in family disconnections.

People don't understand we're a community, you know? We shouldn't be arguing between ourselves. (P9)

The above changes were placed in direct comparison to old ways where (P9) noted ‘everyone used to mix’. Sharing, a strength of Ngarrindjeri ways of doing, was perceived as being eroded. Respect for Elders, especially by the younger generation, was also noted to be in decline.

##### Loss of old ways

Loss of the old Ngarrindjeri ways of knowing, being and doing was tied to the loss of Elders as knowledge keepers:

‘I guess we were hunters and gatherers so uh but that sort of stopped when the I guess, you know, you have someone who leads that in a family. So once they're gone it's sort of lost a little bit’.(P14)

Consequently, the practice of sourcing food guided by the old ways was decreasing, despite a reported abundance of certain produce on Country. The overall decline of traditional food sources further impacted community's ability to practice the old ways.

### 3.3 Currents

Currents were the common threads that flowed like a river throughout the yarns, underscoring both barriers and enablers. Currents were at times explicitly referred to by participants, while at others, discovered as the researcher moved beyond the surface (of the river) in search for deeper meanings.^32^

#### 3.3.1 Colonisation

The ongoing effects of colonisation were reflected in participant experiences of racism, changes to Country and disruption of family, community and food sources. Loss of community and old ways of knowing, being and doing were linked to the introduction into Western society. Moving into previously restricted towns came with access to shops, further experiences of racism and financial dependency:

You sort of lost the old way from the Country, you know, because of the township. And then you got the shops, everything there, they go and buy and if you had your little bit of, um, government money you know, because you would be given money from government, because you – there's no work around, no‐one will employ an Aboriginal person. (P4)

Colonisation and the accompanying changes to Country, legislation and the inaction of physical access barriers, infringed upon accessibility of traditional food sources, while Western food such as white bread, became readily available. At times, participant health was directly and immediately tied to the consumption of Western foods.

Rabbit stew, until the Tip Top man come to my front door and then everyone loved the Tip Top man, then I'm diabetic. (P15)

These participant accounts illustrate how barriers to diabetes care, including experiences in the dominant healthcare system, accessibility and loss, were carried along by the current of ongoing colonisation.

#### 3.3.2 Survival

Despite the continued effects of colonisation, a powerful current of survival underscored major enablers to diabetes care. Participants expressed pride in their identity and joy in connecting and practicing culture. An emphasis on supporting future generations to not only survive but thrive was observed as participants expressed a desire to record, protect and share knowledge of Country and family.

… everywhere I go, I'm really proud of who I am. Just you know, an Aboriginal man… in my culture. (P11)

The continued survival of Ngarrindjeri ways of knowing, being and doing, including a detailed knowledge of Country, privileged participants to work at the interface of Aboriginal and Western knowledge systems. Moreover, keeping culture and community thriving was a recognised driver for ongoing intergenerational knowledge sharing, and motivated participants to take action on diabetes prevalence and mortality.

I keep saying Uncle, that every Elder is a book in our library. And we're losing our books without learning what we could be sharing. (P15)

## 4 DISCUSSION

This study provides valuable insights into the lived experience of Aboriginal people diagnosed with diabetes living on rural Ngarrindjeri Country. From these accounts, it is apparent that participants confront accessibility barriers to quality healthcare and healthy food sources, while experiencing suboptimal care and inadequate knowledge translation in the dominant biomedical healthcare system. Despite these barriers and a profound sense of loss within community, participants continue to express motivation to tackle diabetes management and prevalence. A focus on thriving, not just surviving, for future generations motivates participants throughout the diabetes care journey. Rather than being deprived or disadvantaged, this study suggests that participants are uniquely positioned to address diabetes prevalence and management. Recommendations for diabetes care initiatives are summarised in Table 1.

**TABLE 1:** Recommendations for health promotion interventions with Aboriginal people diagnosed with diabetes on Ngarrindjeri Country.

Key area	Recommendation
Suboptimal care Racism, culturally unsafe care Diabetes knowledge gap	Prioritise community‐led and co‐designed initiatives Improve cultural safety and knowledge translation by employing more AHWs Consider improved cultural safety and health literacy training for GPs on Ngarrindjeri Country Build on existing services provided by local ACCHSs (such as Moorundi ACCHS)
Patient fatigue in dominant Western biomedical system	Avoid activities that centre on biomedical approaches and over‐medicalisation of diabetes care Highlight strengths of Ngarrindjeri ways of knowing, being and doing Consider lifestyle interventions informed by old ways of knowing and being, for example, increased intake of food from Country
Loss	Maintain awareness of the ways loss impacts on community unity, be flexible with intervention implementation Avoid scheduling activities on funeral days Explicitly acknowledge the ongoing effects of colonisation for participants and listen to participant voices on ways to address it
Holistic approach	Incorporate holistic understanding of diabetes care that involves family, Country and community Consider wrap around services that address the social and cultural determinants of health, e.g. provision of transport
Protective factors	Build on, utilise the gift of Two‐Eyed Seeing Utilising community cooking, gardening and leadership skills Tap into community motivation to tackle diabetes (focus on family, community and cultural survival)

Abbreviations: ACCHS, Aboriginal Community Controlled Health Service; AHW, Aboriginal Health Workers.

### 4.1 Addressing barriers

Several barriers identified by participants are reflected in the literature, indicating that parallels may exist between experiences of participants and other Aboriginal communities. Firstly, experiences of suboptimal care in the dominant biomedical system, including encounters with racism, discrimination, or culturally unsafe care are substantial.^6^, ^21^, ^38^, ^39^ Previous research has emphasised the need for culturally safe, community‐led diabetes initiatives to combat these experiences of sub‐optimal care.^6^, ^39^, ^40^ Momentum for these community‐led programs is driven by strong First Nations voices including the Australian Institute of Aboriginal and Torres Strait Islander Studies (AIATSIS),^12^ the National Aboriginal Community Controlled Health Organisation (NACCHO),^41^ as well as a developing body of peer reviewed literature demonstrating their effectiveness on clinical diabetes outcomes and participation rates.^40^, ^42^

Inadequate knowledge translation by healthcare professionals is similarly prevalent in the diabetes discourse,^39^, ^43^ and a recognised barrier to chronic disease service delivery more broadly.^38^ Participants in this study suggested that insufficient time allocated to GPs and high turnover of healthcare staff were contributing factors to the diabetes knowledge gap. Other research has found that diabetes messaging generally is not well understood as it is based on biomedical explanations, rather than holistic Aboriginal conceptions of health.^44^, ^45^ To improve these health literacy outcomes, health promotion efforts should focus on tailoring services to community needs, co‐designing resources with end‐users and increasing the employment of Aboriginal Health Workers (AHWs).^46^ Engaging AHWs is evidenced to improve communication effectiveness and clinical diabetes outcomes in communities with poor access to mainstream services,^40^, ^47^ such as those on Ngarrindjeri Country, and improve the overall diabetes care experience for Aboriginal people.^10^, ^21^, ^38^, ^40^

The emergence of loss as a major barrier was a significant finding. Although existing research has touched upon the relationship between the loss of old ways and the diabetes care journey,^21^, ^43^ this study, to the best of our knowledge, will be the first to identify loss as a major barrier. Any intervention on Ngarrindjeri Country must consider the weight of this loss as well as accompanying practical considerations. For instance, funerals are regularly held at the site of yarn 3 on Thursdays and Fridays.

Finally, although positioned in the context of barriers to diabetes care, patient fatigue and mistrust may equally be understood as an opportunity to implement health promotion activities that shift away from the medicalisation of diabetes care. A history of healthy community and an aptitude for adopting old ways of knowing, being and doing, suggests that Aboriginal people on Ngarrindjeri Country would be receptive to an intervention, such as those proposed by the CDC, that focuses on the strengths and unique benefits of adopting Ngarrindjeri practices.

### 4.2 Holistic view of diabetes care journey

Findings from this study add further weight to appeals by Aboriginal researchers to adopt a holistic approach to health promotion.^14^, ^41^ Participants demonstrated their holistic understanding of diabetes care by centring yarns around Country, culture and family. The interconnectedness of themes and subthemes, as well parallels drawn between the health of Country and the health of community further exemplify this holistic way of knowing and being. Adopting a holistic approach ensures that the Aboriginal concept of health encompassing the physical, psychological, social, spiritual and cultural dimensions of life, is not just recognised but privileged.^48^

Discussions of the social and cultural determinants of health and their relationship to ongoing colonisation, demonstrates the ways participants innately factor these considerations into their holistic approach to the diabetes care journey.^6^ Concerns about transport,^6^, ^21^, ^38^ cost,^39^ socioeconomic factors, quality and access of healthcare,^22^ were all flagged by participants and are reflected in similar qualitative research. By way of the cultural determinants of health, participants reflected on how the dispossession from land and culture had impacted their ability to practice old ways of knowing, being and doing. Considering the use of rations on Ngarrindjeri Country as a means for imposing colonial control, primarily at Point McLeay mission,^49^ it is unsurprising that participants linked their experiences of diabetes care to the forced assimilation into Western society. This sentiment has similarly been found on Wiradjuri Nation, where diabetes has come to symbolise the ‘dispossession, loss and grief associated with colonisation’.^21,p30^

### 4.3 Protective factors

Despite extensive barriers, participants exhibited diverse and strong motivators for addressing diabetes prevalence in community. The ability of participants to adopt the strengths of both Aboriginal and Western knowledge systems, while maintaining pride in Ngarrindjeri community strengths and history of healthy community, helped serve as a protective mechanism. The current of survival, through which participants focused on protecting future generations and intergenerational Ngarrindjeri knowledge sharing, epitomises the strength of community, and sits in direct contrast to the ongoing effects of colonisation.

Lastly, although inadequate knowledge translation was identified as a barrier to diabetes care, participants still exhibited a sound knowledge of Western biomedical approaches. This counterintuitive finding suggests participants were highly motivated to use what little knowledge was at hand to aid self‐management and improve individual and community health. The ability of participants to weave together Aboriginal knowledge of Country, health and food sources, with this Western biomedical knowledge places participants in a unique position to address diabetes prevalence. Possessing the dual understanding of these worlds is a *gift*, as participants utilise the strengths of both worlds to produce novel and positive outcomes for both.^26^, ^50^ Referred to by some as Two‐Eyed Seeing,^50^ this ability mirrors experiences of working at the knowledge interface. The use of plover eggs in cakes is a metaphorical representation of how participants adopted the strengths of both systems, specifically the use of Western and Aboriginal ingredients. The final result, in this case the cake, is a novel finding that was not possible without the ingredients from both systems. This example highlights the ways Aboriginal people on Ngarrindjeri country have matched their worldviews with the contemporary realities of a society dominated by Western ontology and axiology.^26^

Strengths of this study include the adoption of an Aboriginal research methodology and the contribution of Aboriginal researchers to the study design, data collection, interpretation and dissemination of findings at the knowledge interface. A limitation was the small sample size (*n* = 15) as rich data was prioritised over thick data.^37^ The majority of participants were Elders which may influence the generalisability of findings. Future research with the CDC is planned to capture a more diverse subset of community. Although the third yarn saw the repetition of earlier themes, it is believed a fourth yarn would have provided a greater indication of data saturation. Due to a history of being over researched and competing community demands,^18^ additional yarns were not permitted. The researcher responsible for analysis (SOJ) did not attend the yarns as she had no prior links to Ngarrindjeri Country or community. Although this impacted on the ability to obtain observational data, SOJ was mindful that her presence may have impacted the flow, cultural safety and accuracy of the yarns.

## 5 CONCLUSION

Aboriginal people on Ngarrindjeri Country are uniquely positioned to tackle diabetes prevalence by way of a history of healthy community, dual understandings of Western and Aboriginal knowledge systems, diverse motivators, community skills and leadership. Researchers and healthcare providers alike are presented with a notable opportunity to achieve sustainable health equity goals through the co‐design of local solutions, harnessing the potential benefits of this unique position. Findings from this study reinforce the need for such solutions to be holistic, community‐led and privilege Aboriginal ways of knowing, being and doing.

## FUNDING INFORMATION

Funding for this research was obtained as part of a larger Medical Research Future Fund's (MRFF) awarded to the Coorong Diabetes Collaborative (CDC) (2018110).

## CONFLICT OF INTEREST STATEMENT

The authors declare that they have no known competing financial interests or personal relationships that could have appeared to influence the work reported in this paper. The study funders had no role in the study design; collection, management, analysis and interpretation of data; writing of this paper; and the decision to submit this paper for publication. Annabelle Wilson and Courtney Ryder are the Editorial Board members of HPJA and co‐authors of this article. To minimise bias, they were excluded from all editorial decision‐making related to the acceptance of this article for publication.

## ETHICS STATEMENT

Ethics for this research was granted in conjunction with the CDC project from Flinders University (Aboriginal Health Research Ethics Committee—04‐22‐1009, Flinders University Human Research Ethics Committee—5847). NHMRC and AIATSIS guidelines for ethical research with Aboriginal and Torres Strait Islander communities were followed.

## Supporting information


**DATA S1.** Supporting Information.

**DATA S2.** Supporting Information.

## Data Availability

Data used for this research remains the intellectual property of participants and is not available for public access. Research findings belong to the community on Ngarrindjeri country and the CDC. Approval from the CDC Steering Committee was sought prior to publication.

## Endnote

^1^
Terminology adopted for the remainder of this manuscript reflects the research focus with Aboriginal people on Ngarrindjeri Country.
