# Supplementary material for: Exploration of barriers and enablers to diabetes care for Aboriginal people on rural Ngarrindjeri Country
Source: Health Promot J Austr. 2024 Aug 28;36(1):e915. doi: 10.1002/hpja.915 (PMC11730592; doi:10.1002/hpja.915)
Supplement: Supplementary file 2 — DATA S2. Supporting Information. [file HPJA-36-0-s001.pdf]

APPENDICIES

SUPPLEMENTARY Table 1. Methods used by researcher responsible for analysis [blinded for review] to uphold core values for working at the knowledge interface, questions adapted from Durie.[26]

Knowledge Interface Core Value	Question	Methods used to ensure value was upheld
**Mutual respect**	Has the validity of both Western and Aboriginal knowledge systems been recognised / valued in the research?	• Prioritisation of peer received and grey literature by First Nations authors.<br>• Conduct deep listening during discussion with supervisors, informal conversations with Ngarrindjeri academics, community members, examiners, and during analysis of yarn transcripts.<br>• Privileging of Aboriginal voices in final manuscript by way of quotes.<br>• Utilisation of methods derived from both Western knowledge systems (literature review, constructivist grounded theory, thematic analysis) and Aboriginal knowledge systems (Yarning, *Dadirri, Yuri Ingarninthii*).
**Shared benefits**	Have Aboriginal people on Ngarrindjeri Country become active participants in the research? Will they share in the benefits of the research?	• Research need identified by community Elders; spear headed by CDC.<br>• Research outcomes presented to participants by Ngarrindjeri researchers.<br>• Outcomes presented to CDC Steering Committee to check for validity.<br>• Approval of final transcript sought from CDC Steering Committee before publication.<br>• Relevant CDC representatives added to the authorship of the manuscript.<br>• Data remains in ownership of community, is currently being repatriated to community in appropriate mean and mode.<br>• Findings to inform a co-designed diabetes and metabolic intervention on Ngarrindjeri country.
**Human dignity**	Have diverse cultural and spiritual beliefs been valued throughout research without compromise?	• Use of reflexivity journal to document challenges faced if/when diverse cultural/spiritual beliefs did not align with personal beliefs.<br>• Open discussions with supervisors to support the junior researcher through their journey.
**Discovery**	Has a discovery of new knowledge drawn from the two knowledge systems occurred?	• Results analysed using methods form Aboriginal (*Dadirri, Yuri Ingarninthii)* and Western (constructivist grounded theory) knowledge systems enabled the discovery that Aboriginal people on Ngarrindjeri Country are advantaged to address diabetes prevalence and management.

SUPPLEMENTARY Table 2. Key learnings from personal reflexive journey of researcher [blinded for review] in her attempts to avoid extractive nature of research, uphold principles of constructivist approach to grounded theory.

Goal	Actions / Outcomes	What I learnt, felt, did
Avoid extractive nature of research	• Volunteered at South Australian Aboriginal healing festival (*Yarnin Pangari*).	Initially felt uncertain of my place at this festival. Was I just attempting to ease my white guilt? Sweeping floors was not what I had initially envisioned but felt I had a responsibility to contribute.<br>I soon felt welcomed by fellow volunteers and found my place as a small, insignificant spike in the larger wheel to contribute to a successful event. It was a joyful day.<br>I plan on volunteering again next year.
	• Donated funds to Pay the Rent campaign specifying Ngarrindjeri country as location.	I had donated to this campaign before but recognised the need to increase my efforts. I was initially annoyed the payment was not tax deductable. Upon reflection, I recognised my unconscious tendency to want to "extract" something (i.e. a tax deduction) from the process.
Develop deep listening *(Dadirri/ Yuri Ingarninthii)* skills	• Purchased and read Ngarrindjeri publications.[29, 49†]<br>• Engaged in informal discussions with Ngarrindjeri community members and academics.	I observed myself being emotionally impacted by continued recounts of trauma in some of these publications. As a white woman, I am privileged with the ability to 'switch off' and separate my experiences from those recorded in these publications.<br>I was disappointed, but not surprised, that my learnings to date had not covered the rich history of the Country adjacent to Kaurna Yerta (my birth place). I have since shared my learnings with work colleagues and in informal conversations with family/friends.<br>In what has come to mirror my findings, continued readings and informal interactions with Ngarrindjeri people led me to recognise the sheer strength and resilience of Ngarrindjeri people.

[†]Additional publications not directly sighted in this manuscript include:

- Bell D. Ngarrindjeri Wurruwarrin: A world that is, was, and will be. 2 ed. Melbourne VIC: Spinifex Press; 2014.
- Ngarrindjeri Lands and Progress Association Inc., Bell D, editor. Kungan Ngarrindjeri Miminar Yunnan. Melbourne VIC: Spinifex Press; 2008.
- Rochester A. Ngarrindjeri Ways of Being, Doing and Knowing: A Classroom Reflection on Relationality. Adelaide: Flinders University of South Australia; 2019.

		Deep listening is a skill and requires practice. I hope to continue developing this skill, mindful it will take time.
Redress power imbalances	• Positioning myself as a 'trainee' learning from the experiences of participants.	Throughout this entire journey I have sought to position myself as learner, while my supervisors, the CDC Steering Committee, and community were thought of as teachers. I have a strong desire to continue these learnings as I am certain I have barely scratched the surface. I feel a sense of responsibility to transform these learnings into actionable outcomes that will benefit community.
Generate sense of reciprocity	• Plans in place with CDC Steering Committee to meet and develop relationships with community.	I feel nervous about bridging this gap but recognise how this will ensure a sense of accountability for me as a researcher and as a person.<br>I am drawing on my past experiences of relocating to Southeast Asia during my formative years, where I was exposed to different epistemologies and ontologies, to help guide me on this process. I am acutely aware the profound sense of joy such a self-reflexive, challenging and eye-opening experience can bring.
Reflect on my unconscious biases as a researcher	• Reflexivity journal.	Observed my own tendency to view systems, objects, bodies as compartmentalised and placed into categories. I began to see the interrelatedness or relationality of all things and developed an increased sense of responsibility, to do right by participants and accurately represent their lived experiences. My eureka moment occurred when I learnt that the Ngarrindjeri plural of body (ruwar) was Country (Ruwi). To me, this epitomised the relationality of all living things. People are Country. Country is people.
Reflect on how standpoint influenced data generation		Due to my absence from the yarns, the impact of my standpoint was limited to data analysis and excluded data generation.
